# Safety and Cost-Effectiveness of Interscalene Brachial Plexus Block With Sedation in Reverse Total Shoulder Replacement

**DOI:** 10.7759/cureus.14106

**Published:** 2021-03-25

**Authors:** Kiran Ramesh, Muhammad Yusuf, Navnit Makaram, Ross Milton, Aji Mathew, Makaram Srinivasan

**Affiliations:** 1 Orthopedics and Traumatology, Wirral Teaching Hospitals NHS Trust, Wirral, GBR; 2 Orthopedics and Traumatology, Pennine Acute Hospitals NHS Trust, Manchester, GBR; 3 Orthopedics and Traumatology, Royal Infirmary of Edinburgh, Edinburgh, GBR; 4 Anaesthesiology, Lancashire Teaching Hospitals NHS Trust, Preston, GBR; 5 Anaesthesia, East Lancashire Hospitals NHS Trust, Blackburn, GBR; 6 Orthopedics and Traumatology, East Lancashire Hospitals NHS Trust, Blackburn, GBR

**Keywords:** inter-scalene brachial plexus block, regional anaesthesia, reverse total shoulder replacement, cost effectiveness

## Abstract

Aims

To investigate the safety and cost-effectiveness of interscalene brachial plexus block/regional anaesthesia (ISB-RA) in patients undergoing reverse total shoulder replacement.

Patients and methods

This retrospective study included 15 patients with symptomatic rotator cuff arthropathy who underwent reverse total shoulder arthroplasty (rTSA) under ISB-RA without general anaesthesia in the beach chair position from 2010 to 2018. The mean patient age was 77 years (range 59-82 years). Patients had associated medical comorbidities: American Society of Anesthesiologists (ASA) grade 2-4. Assessed parameters were: duration of anaesthesia, intra-operative systolic blood pressure variation, sedation and vasopressor use, duration of post-operative recovery, recovery scores, length of stay, and complications. A robust cost analysis was also performed.

Results

The mean (range) duration of anaesthesia was 38.66 (20-60) min. Maximum and minimum intra-operative systolic blood pressure ranges were 130-210 and 75-145 mmHg, respectively (mean [range] drop, 74.13 [33-125] mmHg). Mean (range) propofol dose was 1.74 (1-3.0) mg/kg/h. The Median (interquartile range) post-operative recovery time was 30 (20-50) min. The mean (range) postoperative recovery score (local scale, range 5-28 where lower values are superior) was 5.2 (5-8). The mean (range) length of stay was 8 (1-20 days); the two included patients with ASA grade 2 were both discharged within 24 hours. One patient with predisposing history developed pneumonia; however, there were no complications related to ISB-RA. The mean (range) cost per patient was £101.36 (£59.80-£132.20).

Conclusions

Our data demonstrate that rTSA under ISB-RA is safe, comfortable, and cost-effective. Notably, patients with ASA grade 2 who underwent rTSA under ISB-RA had a reduced length of stay and were discharged within 24 hours.

Clinical relevance

rTSA under ISB-RA is potentially a safe, cost-effective, and viable alternative for patients with multiple comorbidities.

## Introduction

Interscalene brachial plexus block/regional anaesthesia (ISB-RA) has revolutionised same-day upper limb surgery procedures and become a standard anaesthetic technique in shoulder surgery, as an alternative to, or sometimes in combination with, general anaesthesia (GA).

Regional anaesthetic (RA) techniques are safe, with excellent patient acceptance and shorter hospital stays, compared with GA, in arthroscopic shoulder procedures [1]. Further, using RA in conjunction with GA can reduce the GA dose and provide variable duration postoperative analgesia. While the literature is replete with articles on the use of ISB-RA in shoulder arthroscopy [1] and its effectiveness as a postoperative analgesic, there is scarce information on its use alone, as the sole anaesthetic, in patients undergoing open shoulder procedures, such as shoulder replacement surgery.

Most patients undergoing reverse total shoulder replacement surgery in the UK are in their sixth or seventh decade of life and many have significant cardiac and pulmonary comorbidities, making GA a high-risk procedure. Here, we investigated clinical outcomes and cost-effectiveness of ISB-RA with sedation, as an alternative to GA combined with a similar block, in 15 patients who underwent reverse shoulder replacement.

## Materials and methods

The study protocol was approved by the responsible local institutional clinical audit and clinical effectiveness committee.

Patients

**Figure 1:**
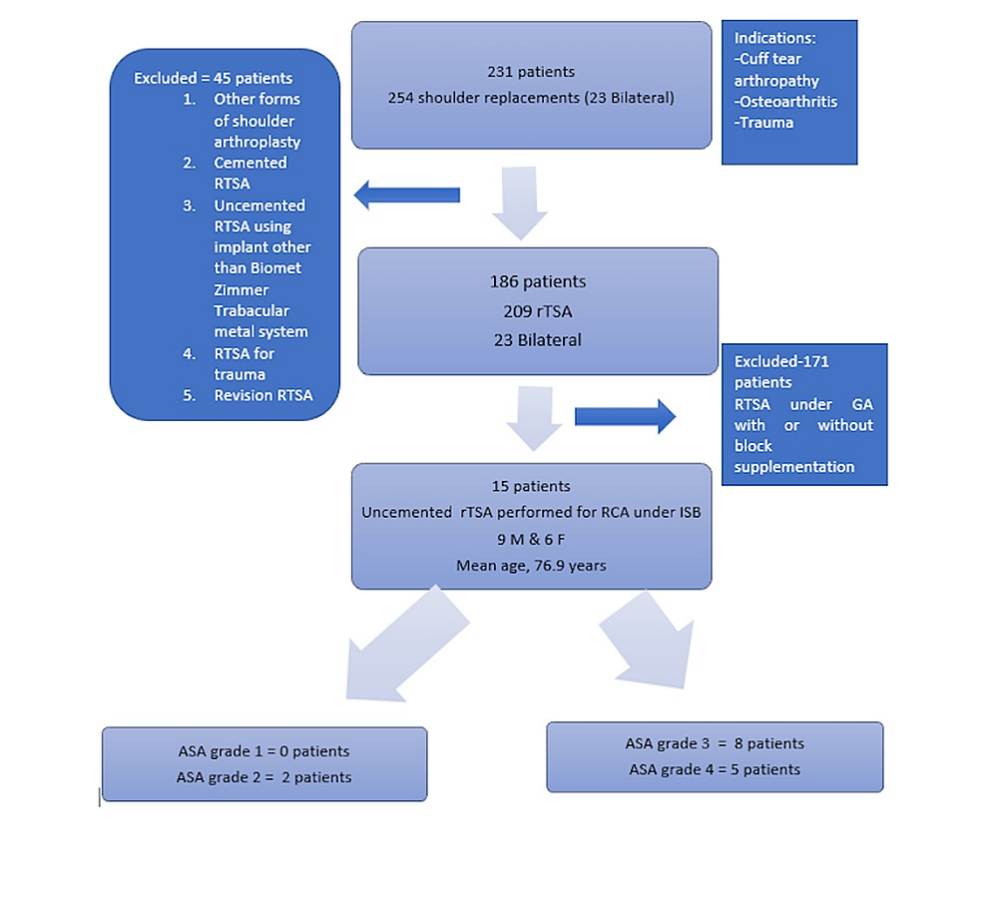
Flowchart rTSA/RTSA - reverse total shoulder arthroplasty; RCA - rotator cuff tear arthropathy; ISB - interscalene block; GA - general anaesthesia; M - male; F - female; ASA - American Society of Anesthesiologists

A database collected from 2010 to 2018 was reviewed to identify patients who underwent reverse total shoulder replacement surgery using the Zimmer Biomet Trabecular Metal cement-free system (Zimmer, Inc., IN, USA) under ISB-RA, supplemented with sedation (Figure 1).

Patient inclusion and exclusion criteria are detailed in Table 1.

**Table 1:** Inclusion and exclusion criteria rTSA - reverse total shoulder arthroplasty; GA - general anaesthetic; RA - regional anaesthetic; ASA - American Society of Anesthesiologists

Inclusion criteria	Exclusion criteria
rTSA	Other forms of shoulder arthroplasty: hemiarthroplasty, total shoulder replacement, and shoulder resurfacing hemiarthroplasty
Uncemented rTSA using the Biomet Zimmer Trabecular Metal system	Cemented rTSA and uncemented rTSA using implant other than the Biomet Zimmer Trabecular Metal system
rTSA performed under RA and supplemental sedation	rTSA performed under GA or a combination of general and RA
rTSA performed electively for symptomatic rotator cuff tear arthropathy	rTSA performed for trauma (unreconstructable proximal humerus fractures), primary or secondary malignancy, or for revision of failed shoulder arthroplasty
Patients with significant co-morbidities (ASA 3–4) and patients with ASA 2 who opted for regional block.	ASA 3–4 patients who were fit for GA and ASA 1–2 patients who did not consent to regional block anaesthesia alone

Only patients who underwent elective uncemented reverse total shoulder arthroplasty (rTSA) for symptomatic rotator cuff tear arthropathy, using the same implant by a single senior consultant orthopaedic shoulder surgeon, were included. Patients with other forms of shoulder arthroplasty and indications other than symptomatic rotator cuff tear arthropathy were excluded. Patients were assessed pre-operatively by the senior consultant anaesthetist and, following a discussion regarding the risks and rewards, patients with American Society of Anesthesiologists (ASA) grade 3 and 4 comorbidities, who consented to undergo the surgical procedure under ISB-RA supplemented with conscious sedation, were included. Two patients with ASA grade 2 who requested that the procedure be performed under RA alone were also included in the study. Fifteen patients were identified who satisfied the inclusion criteria between December 2010 and November 2018 (Figure 1); they had excessive uncontrollable pain due to rotator cuff tear arthropathy that had failed non-operative management procedures, such as activity modification, local infiltration, and highest pain management regimen. Patients were medically optimised and, on occasion, surgery in patients with respiratory comorbidities was deferred until the summer for the best chance of recovery. 

Patient data

The database recorded information on demographics, surgical indications, type of anaesthesia, implanted rTSA component sizes, intra- and post-operative complications, and radiographic findings on 6-month, 12-month, and yearly follow-up radiographs. All data were recorded by fellowship-trained senior shoulder surgeons. Radiographic assessments were performed by the senior author and independently validated by a musculoskeletal radiologist and two specialist shoulder registrars. 

Information regarding the duration of RA was obtained from Theatreman theatre management software (Trisoft, Nottingham, UK). Intra-operative vital parameters, sedation and vasopressor use, duration in post-op recovery, and recovery scores were recorded from anaesthetic charts. Postoperative recovery scores devised locally at our hospital were used, range 5 to 28, with lower values indicating superior patient parameters. Post-operative complications and length of stay (LOS) were from patient case notes. 

Ultrasound-guided interscalene block procedure

ISB-RA was performed by a fellowship-trained senior consultant anaesthetist in the anaesthetic room with the patient in a semi-sitting position.

The linear transducer (8-14 MHz) was positioned in the transverse plane (at the supraclavicular fossa) to locate the scalenus anterior and medius muscles. The C5 and C6 roots were identified and the probe moved a few millimetres above the point at which the suprascapular nerve branched off from the C5 root. After infiltrating the skin at the entry point under sternocleidomastoid with 5 mL of 1% lidocaine using a 27 G hypodermic needle, a 50 mm echogenic needle (Sonoplex®, Pajunk, Geisingen, Germany) was inserted using an in-plane technique from a lateral to medial direction (close to the C5 and C6 nerve roots) and aimed towards the brachial plexus nerve roots. 

After identifying a ‘pop’ following penetration of the needle through the pre-vertebral fascia, the needle was advanced between the fascia and middle scalene muscle towards the brachial plexus. A small amount of 2% lidocaine was injected. Inline needle visualisation ensured adequate spread of the local anaesthetic around the plexus, with the displacement of the plexus away from the needle. A total of 10 mL of 2% lidocaine was infiltrated around C5 and C6, followed by 15-20 mL of 0.25%-0.5% levobupivacaine (depending on the weight of the patient) to cover the entire brachial plexus (including C7, C8, and T1, to form a ‘halo’ around the plexus).

After confirming the adequacy of the C5 and C6 dermatomal block, target-controlled infusion (TCI) sedation with 1% propofol was started to achieve plasma concentrations of 1-2 µg/ml (Marsh model), and oxygen was delivered through a Hudson mask. Intraoperatively, the TCI dose was altered to achieve the desired depth of sedation and was stopped after surgery.

Surgical procedure

Surgery was performed in the beach chair position. A modified antero-superior deltoid split approach was used, as previously described by Molé et al. [2] with conservative acromioplasty and excision of 5-10 mm of the lateral end of the clavicle. The rTSA was implanted, cement-free, according to the manufacturer’s recommendations. 

Cost analysis

The cost analysis considered consumables (anaesthetic drugs), disposables, and personnel costs, including cost per consultant anaesthetist. As continuous peripheral nerve catheters were not used, we considered the cost of only one anaesthetic consultant. As all blocks were administered in the anaesthetic room, theatre time cost was not included. Information regarding drug costs was obtained from the British National Formulary (BNF), and that for consumables from the theatre supply chain department at our hospital. Drug cost calculations assumed that residual drugs in an ampoule were discarded. The cost per consultant anaesthetist was based on data from the Personal Social Services Research Unit [3].

## Results

Patient characteristics

The 15 included patients comprised nine females and six males; the mean (range) age was 76.9 (59-82) years (Table 2).

**Table 2:** Patient demographics

Age (Average)	76.9
Age Subgroups	
50-60	1
60-70	3
70-80	8
80-90	3
Gender	
Female	9
Male	6

Patients had ASA grades ranging from 2-4 (Figure 1). Detailed information on patient comorbidities is provided in Table 3.

**Table 3:** Co-morbidities and ASA grade ASA - American Society of Anesthesiologists; GFR - glomerular filtration rate; MI - myocardial infarction; LVF - left ventricular failure; CCF - congestive cardiac failure; AF - atrial fibrillation; COPD - chronic obstructive pulmonary disease; CPAP - continuous positive airway pressure; SOB - shortness of breath; EF - ejection fraction; CKD - chronic kidney disease; CABG - coronary artery bypass grafting; NIDDM - non-insulin-dependent diabetes mellitus; TIA - transient ischaemic attack

Patient No.	ASA grade	Comorbidities
1	4	Dilated cardiomyopathy, pacemaker, GFR 15–20, MI, LVF, CCF, AF on warfarin
2	4	Previous MI (1995), angioplasty (1996), paroxysmal AF, COPD
3	4	Bronchiectasis: severe cystic change, empyema, tracheomalacia. Previously on CPAP
4	4	Severe COPD, MI, epilepsy, SOB on exertion, central cyanosis, EF of 35%
5	4	Hypertension, CKD five on dialysis awaiting transplant, smoker 10/day, vasculitis
6	3	Bronchial asthma, type 2 diabetes
7	3	MI, CABG 2008, alcoholic liver disease
8	3	Ischaemic heart disease, sleep apnoea on CPAP
9	3	Moderate aortic stenosis, ischaemic heart disease. Angioplasty (2012). Mild disease: childhood asthma
10	3	Atrial fibrillation, orthopnoea, ischaemic heart disease, mild Cvf
11	3	Hypertension, lobectomy 2009, NIDDM, nephrectomy for renal cancer
12	3	Hypertension, diabetes, TIA
13	3	TIA, COPD, CABG -2000, epilepsy, CKD
14	2	TIA
15	2	Hypertension

There was no patient mortality at 30-day or one-year follow-up. 

Duration of anaesthesia

The mean duration of RA, measured from patient arrival in the anaesthetic room to entry into the operating theatre, accounting for the time to perform the block and for the block to take effect, was 38.66 min (range, 20-60 min) (Table 4).

**Table 4:** Clinical characteristics BP - blood pressure; LOS - length of stay

Parameter	Patient															Mean/median
	1	2	3	4	5	6	7	8	9	10	11	12	13	14	15	Mean
Anaesthesia time (min)	60	50	30	30	40	30	20	25	45	35	40	45	60	40	30	38.66
Systolic BP Drop (mm Hg)	80	75	50	90	33	65	100	50	85	105	125	80	70	54	50	74.13
Propofol µg/ml	2	2	2	1.4	2	2	1.7	1.3	1.6	3	1	2	1.5	1.3	1.3	1.74
Recovery time (Min)	50	75	40	20	80	20	20	45	30	50	240	25	40	25	45	30 (Median)
LOS (Days)	14	5	20	8	6	3	3	2	18	15	17	5	2	1	1	8

Systolic blood pressure variations 

The highest systolic blood pressure ranged from 130-210 mmHg, while the minimum systolic blood pressure was 75-145 mmHg, with a mean (range) drop of 74.13 (33-125) mmHg (Table 4).

Sedation and vasopressor requirements

Mean (range) propofol plasma concentration for sedation using target-controlled infusion was 1.74 (1-3.0) µg/ml. Three patients, all of whom were on beta-blockers, received three doses (1.5 mg) of metaraminol each (Table 4).

Recovery time

One patient with a history of lung lobectomy for metastasis and nephrectomy for renal cancer had an extended (planned) stay in recovery of 240 min. The median (interquartile range) for recovery time in the post-anaesthetic care unit was 30 (20-50) min (Table 4).

Recovery score

The criteria for discharge during recovery included: score < 10; temperature 36-37ºC; and no individual score > 2. All patients showed significant improvement during recovery (mean score = 5.2; range, 5-8).

Complications

One patient with a history of chronic obstructive pulmonary disease (COPD), paroxysmal atrial fibrillation, and myocardial infarction developed hospital-acquired pneumonia. There were no complications related to the interscalene block.

Length of stay

Mean (range) LOS was 8 (1-20) days; this was extended in one patient due to medical complications. Four patients had underlying cardiac, pulmonary, and renal problems that each required multi-disciplinary input and four patients stayed for longer because they were awaiting a care package. Both patients with ASA grade 2 were discharged within 24 hours of surgery (Table 4).

Cost analysis

Unit costs of consumables are presented in Table 5.

**Table 5:** Unit costs

Item	Unit cost (£)	Source
Plasmalyte	7.00	NHS Hospital
Giving set	0.79	NHS Hospital
Extension set	1.90	NHS Hospital
Wound care	0.46	NHS Hospital
Ultrasound gel	1.05	NHS Hospital
Nerve needle	13.10	NHS Hospital
Tegaderm	0.12	NHS Hospital
Chloroprep	0.81	NHS Hospital
Pink Venflon	0.51	NHS Hospital
Syringe (50 ml)	0.51	NHS Hospital
Syringe (20 ml)	0.07	NHS Hospital
Syringe (10 ml)	0.03	NHS Hospital
Syringe (5 ml)	0.02	NHS Hospital
Safety needle	0.08	NHS Hospital
Probe cover	1.07	NHS Hospital
Lidocaine 2% 10 ml × 2	0.64	BNF
0.5% Levobupivacaine 10 ml × 2	3.23	BNF
Total cost of consumables & disposables	31.39	

The cost per consultant anaesthetist was £1.81/min. Based on the mean anaesthesia time of 38.66 min, the mean (range) cost per patient was £101.36 (£59.80-£132.20) (Table 6).

**Table 6:** Cost analysis per patient RA - regional anaesthesia

Patient No.	Duration of RA (min)	Cost excluding theatre time (£)
1	60	139.99
2	50	121.89
3	30	85.69
4	30	85.69
5	40	103.79
6	30	85.69
7	20	67.59
8	25	76.64
9	45	112.84
10	35	94.74
11	40	103.79
12	45	112.84
13	60	139.99
14	40	103.79
15	30	85.69
Average	38.66	101.36

## Discussion

Reverse shoulder replacements are currently the most common replacement shoulder arthroplasties performed in the UK in both the National Health Service and the independent sector. Such procedures provide consistent pain relief, combined with good shoulder mobility, resulting in significant improvements in life quality for older patients with advanced rotator cuff tear arthropathy. 

GA alone is associated with high postoperative pain scores and readmission rates, and longer hospitalisation, necessitating the addition of some form of RA [4]. Complications and mortality risk with GA alone increase with age and associated co-morbidities. Interscalene block is effective for analgesia in shoulder surgery [5], and supplementation with GA, to achieve good postoperative analgesia is standard practice in most centres; however, there is scant evidence for its use as a sole anaesthetic in reverse shoulder arthroplasty. 

No difference in pain score or morphine consumption was reported between patients in a randomised control trial receiving low versus standard ropivacaine dose [6]. Although we observed excellent anaesthesia with as little as 5 mL of 0.5% bupivacaine, we used the standard dose to minimise postoperative opioid use. The number of needle passes were also kept to a minimum, and we encountered no problems with intravascular infiltration with the use of ultrasound guidance. No patients experienced any systemic symptoms. Reported conversion rates from RA to GA are 8.7% to 13% [5]. No patients in our series had failed or inadequate block requiring intra-operative conversion to GA.

In a non-randomised retrospective cohort analysis, comparing RA versus GA in patients who had undergone a total and reverse shoulder replacements, rTSA accounted for 35% of shoulder replacements, and 16.98% underwent the rTSA procedure under RA, with 9.1% of patients experiencing in-hospital post-operative complications, which was not significantly different from the GA group [7]; however, respiratory in-hospital complications were more significant in the GA than the RA group. In our series, one patient (6.6%) had an in-hospital complication. Further, in the previous study, mean LOS in the GA group (two days) was significantly shorter than that of the RA group (2.3 days) [7], which compares favourably with the mean LOS in our series (eight days); however, in the previous series, 61.01% of patients had two and three co-morbidities according to Elixhauser stratification, whereas such patients accounted for 86.6% in our study. The average LOS of eight days in our study also reflects the physiological fitness levels of the patients under evaluation, most of whom had ASA grade 3 or 4. Notably, the two patients with ASA grade 2 were discharged the following day, an important consideration in the move towards same-day shoulder replacement surgery. Moreover, the previous study had a 4.2% 90-day readmission rate in the RA group, while none of our patients required re-admission [7].

GA can have a profound effect on cerebral perfusion in patients with relative hypotension placed in the beach chair position. An 80% incidence of cerebral desaturation events was reported with GA in the beach chair position, associated with postoperative nausea and vomiting [8]. Significantly higher incidence rates of postoperative pneumonia, prolonged ventilator dependence, and unplanned postoperative intubation were found in a subset of COPD patients undergoing surgery under GA compared with RA [9]. RA maintains cerebral autoregulation more effectively and has a less profound effect on cerebral perfusion than GA [10]. Yadeau et al. [11] used near-infrared spectroscopy in patients undergoing shoulder surgery under RA in the beach chair position and reported a 77% incidence of hypotension but only 0.77% incidence of cerebral desaturation events. In our series, systolic blood pressure was maintained, with a mean drop of 74 mmHg, which was significant, despite the sympathetic vasomotor system being unaffected by the block. The preservation of this vital autonomic function is important to minimize risk to the patient. In our series, three patients required vasopressor support, with a maximum total dose of 1.5 mg metaraminol. All three patients had moderate to severe cardiovascular disease and had been on medications (beta-blockers, angiotensin-converting enzyme inhibitors, or angiotensin receptor blockers) that increased their susceptibility to intraoperative hypotension. No patient required postoperative cardiovascular support. Intraoperative or immediate postoperative stroke is rare when surgery is performed in the beach chair position under RA, with propofol sedation, and spontaneous respiration through a natural airway [12]. Although five patients in our series had prolonged stays, these were due to pre-existing co-morbidities and not related to the ISB-RA.

Primary rTSA for rotator cuff arthropathy has modest-cost effectiveness, based on EuroQual and Short Form 36 analyses [13]. The anaesthetic-related costs of surgery under brachial plexus block were significantly less than those under GA [14]. Comparison of patients who underwent ambulatory total shoulder arthroplasty with a matched group undergoing in-patient shoulder arthroplasty demonstrated significantly lower costs in the former group, with no difference in the complications and readmission rates between the two groups [15]. Further, a comparison of economic aspects of ultrasound-guided ISB-RA with GA for arthroscopic shoulder procedures found that the total cost per case for ISB-RA was significantly lower than that of GA alone [16]. That study excluded patients with ASA grade 4, did not consider personnel costs, and estimated the total cost per case at 33 Euros for the ISB-RA group, which is comparable to our findings (total cost per case, £31.39). 

ISB-RA is not without complications. The incidence of ipsilateral phrenic nerve palsy varies from 25% to 100% [6,17], and can lead to a 25% decline in lung function, with the diaphragmatic paresis compensated for by the intercostals and accessory respiratory muscles; hence, RA should be used with caution in patients with compromised lung function. All patients in the current study were managed with this expectation postoperatively. Consistent with the findings of a randomised study comparing RA versus GA in patients who underwent shoulder arthroscopy, which found no requirement for oxygen at discharge from recovery in the RA group, compared with 59% of patients in the GA group that did require oxygen [18], none of our patients, including three who had severe respiratory disorders preoperatively, required extended respiratory support beyond supplemental oxygen. 

Pneumothorax is rare. Blockade of the cervical sympathetic nerves causes Horner’s syndrome. Recurrent laryngeal nerve blockade can lead to hoarseness and complete airway obstruction in subjects with pre-existing vocal cord paralysis. In addition, the Bezold-Jarisch reflex [19],^ ^characterised by hypotension, bradycardia, and coronary vasodilatation, due to vagal afferent stimulation, in response to increased levels of circulating catecholamines, should be considered, particularly during shoulder surgery in the beach chair position; incidence is reported as 15-30% and treatment consists of volume replacement, atropine, and ephedrine. In our series, transient hypotension was effectively managed using low doses of metaraminol and was more likely the consequence of preoperative medication. Other rare complications include permanent neurological injury and accidental injection to epidural, subarachnoid space, or vertebral artery injection, which can be prevented by good technique. None of these complications were encountered in our series.

Our study had many limitations. It was retrospective with low patient numbers. Further, as an observational case series, there was a lack of controls for comparison. The cost analysis included only fixed costs; time analysis related to the anaesthesia workflow was not conducted. Nonetheless, our observations demonstrate that ISB-RA, supplemented with sedation, is clinically effective and safe in high-risk (ASA 4) patients and has low complication rates. The findings concurred with National Institute for Health and Care Excellence (NICE) guidelines (NG 157) evidence review of anaesthesia in shoulder surgery [20], which found RA beneficial in terms of re-admission and considered the possibility of faster, same day discharge.

## Conclusions

ISB-RA supplemented with sedation, for patients undergoing reverse shoulder replacement in beach chair position generated favourable results in our series of high-risk patients, in terms of acceptability, safety, complication rates, and cost-effectiveness. Patients who may otherwise have been considered unfit for surgery underwent the procedure safely under the block, with an expectation of improving their functionality and quality of life.

However, high-quality studies comparing RA versus GA in shoulder arthroplasty are required in the future to investigate clinical outcomes and cost-effectiveness in patients of all risk groups undergoing shoulder replacement, as well as the addition of enhanced recovery, to explore the possibility of offering shoulder replacement surgery under RA as a day care procedure.
